# Development and Evaluation of Clinical Practice Guidelines for Patients Undergoing Hepatectomy

**DOI:** 10.3390/healthcare14131939

**Published:** 2026-07-01

**Authors:** Orathai Kaewjaladvilai, Suchira Chaiviboontham, Bualuang Sumdaengrit, Pakkapol Sukhvibul, Thamonwan Yodkolkij

**Affiliations:** 1 Ramathibodi School of Nursing, Faculty of Medicine Ramathibodi Hospital, Mahidol University, Bangkok 10400, Thailand; orathai.k@nmu.ac.th (O.K.); bualuang.sum@mahidol.ac.th (B.S.); 2 Department of Surgery, Faculty of Medicine Vajira Hospital, Navamindradhiraj University, Bangkok 10300, Thailand; pakkapol@nmu.ac.th; 3 Nursing Division, Faculty of Medicine Vajira Hospital, Navamindradhiraj University, Bangkok 10300, Thailand; thamonwan.yo@nmu.ac.th

**Keywords:** clinical guideline development, ERAS protocol, feasibility study, hepatectomy liver cancer patients

## Abstract

**Background**: Liver cancer is a major public health problem in Thailand due to its high incidence and mortality. Although hepatectomy is a potentially curative treatment, it is a complex procedure with a high risk of postoperative complications, necessitating a structured and systematic approach to care. **Objectives**: This study aimed to develop a clinical practice guideline (CPG) for patients with liver cancer undergoing hepatectomy and to evaluate the feasibility of its implementation in relation to outcomes for healthcare providers, the organization, and patients. **Methods**: This implementation research was conducted in three phases: (1) an evidence-triggered phase, (2) an evidence-supported phase, and (3) an evidence-observed phase. The CPG covered five stages of care: preoperative, intraoperative, postoperative, discharge planning, and post-discharge follow-up. It was implemented through a multidisciplinary approach, with an advanced practice nurse (APN) facilitating adherence to Enhanced Recovery After Surgery (ERAS) components. Data were analyzed using descriptive statistics. **Results**: Healthcare personnel demonstrated high adherence to the CPG and reported high feasibility of implementation. After implementation, favorable trends were observed in postoperative complications, length of hospital stay, hospitalization costs, and patient satisfaction compared with the historical pre-implementation period. The CPG also appeared to support clearer care standards and multidisciplinary coordination. **Conclusions**: The developed CPG was feasible and contextually appropriate for ERAS-based hepatectomy care in this setting. Preliminary findings suggest favorable trends in care processes and selected outcomes. Larger controlled studies with longer follow-up are needed to determine effectiveness and sustainability.

## 1. Introduction

Hepatectomy is the best curative option in patients with appropriate liver function (Child–Pugh class A/B). However, hepatectomy is a major operation involving a complex of hepatic anatomy and vasculature, prolonged operative time, and the risk of substantial blood loss and tissue injury, resulting in a high incidence of postoperative complications [1,2]. The incidence of complications after hepatectomy has been reported to range from 30% to 45% [3]. The most frequent complication is post-hepatectomy liver failure (PHLF), with reported incidence varying between 1.2% and 32% [4,5], post-hepatectomy ascites 7.2% [6], bile leakage 4–17% [7,8], postoperative infections (catheter-related, cardiopulmonary, intra-abdominal, urinary tract, and surgical site infection (SSI)) 30% [7], and post-hepatectomy hemorrhage (PHH) 3% [9]. These complications are associated with delayed postoperative recovery, increased length of hospital stay (LOS) and healthcare costs, and a decreased quality of life [10].

In the implementation setting, at the University Hospital, the number of patients with liver cancer undergoing hepatectomy has continued to increase. In 2023, 87 patients underwent hepatectomy, compared with 48, 54, 58, and 82 cases in 2019–2022, respectively. However, a clear clinical practice guideline (CPG) has not yet been established, resulting in care that largely depends on individual experience and leading to variability in monitoring surgery-specific risks. In addition, there is no systematic model for providing self-care education to patients and their caregivers. Therefore, there is a system-level need to develop a CPG for patients with liver cancer undergoing hepatectomy by applying the Enhanced Recovery After Surgery (ERAS) Society Recommendations 2022 [11] within a multidisciplinary care model. The goal is to reduce postoperative complications, length of stay, readmissions, and healthcare costs, with advanced practice nurse (APN) serving as a key implementation mechanism to evidence-based care [12].

ERAS is a perioperative care pathway designed to reduce the surgical stress response and has been shown to reduce postoperative complications and LOS across multiple surgical procedures [13,14,15]. The Guidelines for Perioperative Care for Liver Surgery: Enhanced Recovery After Surgery (ERAS) Society Recommendations 2022 categorize perioperative management into three phases: preoperative, intraoperative, and postoperative care [11]. In some settings, ERAS implementation may remain largely physician-directed and may lack concrete multidisciplinary practice guidance. This suggests limited multidisciplinary adherence to ERAS-based care recommendations [16].

In terms of CPG development and implementation, a literature review found that the importance of an evidence-based practice framework and application science are essential for reducing the gap between research evidence and routine practice [17,18,19,20]. Accordingly, evaluation of CPGs should encompass guideline quality, the implementation process, and implementation outcomes within real-world contexts [21,22,23], as well as the perceived feasibility of guideline implementation by personnel, to support continuous improvement and development [24,25]. Regarding the role of APN, they play a crucial role in linking evidence to practice through the care of patients with complex health problems, the management and supervision of specialized patient care systems, the coordination of multidisciplinary teams, clinical counseling, and the empowerment of patients and personnel. This includes change agents in the development and implementation of CPG within an organizational context [12]. This role is consistent with the development of practice guidelines for patients with liver cancer undergoing hepatectomy, which requires clinical expertise, systemic management, and continuous outcome monitoring and evaluation.

**Figure 1:**
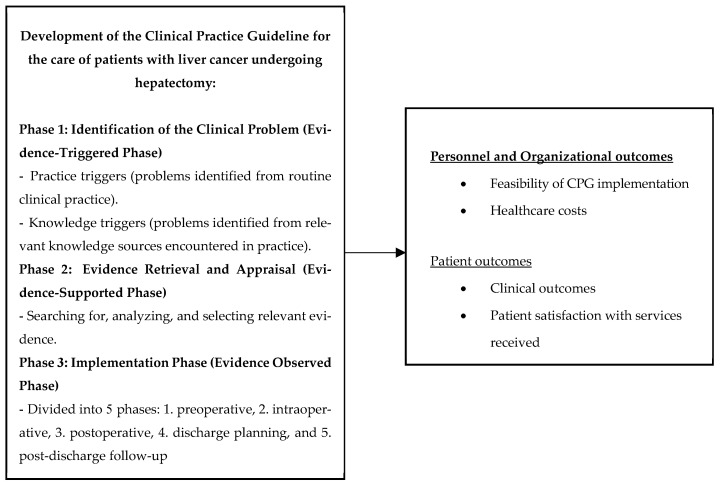
Framework for CPG development.

Therefore, the researchers recognized the need to develop a CPG for the care of patients with liver cancer undergoing hepatectomy by integrating the ERAS Society Recommendations 2022 with evidence-based practices. This study utilized Soukup’s [19] evidence-based nursing practice guideline development model, which features a clear process. In this study, it consisted of three phases: (1) evidence-triggered phase, (2) evidence-supported phase, and (3) evidence-observed phase. The evidence-triggered phase was used to identify clinical problems and practice needs; the evidence-supported phase was used to search, appraise, and synthesize evidence for CPG development; and the evidence-observed phase was used to implement and evaluate the developed CPG in clinical practice. To ensure a systematic implementation process, the study applied the implementation outcome framework proposed by Proctor et al. [25] combined with multi-component implementation strategies, including educational meetings, to establish shared understanding and strengthen multidisciplinary team readiness, provision of educational materials, and continuous monitoring, evaluation, and feedback [26]. Advanced practice nurses served as the primary implementation drivers by synthesizing evidence and facilitating multidisciplinary implementation appropriate to the organizational context. The outcome variables were categorized in accordance with the framework for CPG development. Personnel and organizational outcomes included feasibility of CPG implementation and healthcare costs, representing implementation and organizational/service outcomes. Patient outcomes included clinical outcomes and patient satisfaction with services. Clinical outcomes comprised postoperative complications, length of hospital stay, postoperative recovery indicators, and 28-day readmission. The developed practices were intended to support standardized care, multidisciplinary coordination, and systematic monitoring of key clinical and service outcomes. The framework for CPG development is presented in Figure 1.

## 2. Materials and Methods

### 2.1. Study Design

This implementation research aimed to develop and evaluate the feasibility of a CPG for patients undergoing hepatectomy. The study was conducted at the surgical clinic and inpatient wards of the surgical department of a University Hospital during March to July 2025, with approval from the Human Research Ethics Committee.

Phase 1: Evidence-triggered phase. Clinical problems were identified from two sources: (1) practice triggers, which indicated that no clear guideline existed for caring for patients with liver cancer undergoing hepatectomy. Practices remain routine and variable, and specific risk monitoring is incomplete; and (2) knowledge triggers, whereby the literature review indicated limited evidence on comprehensive, systematic care spanning the preoperative period through post-discharge follow-up. In addition, many studies on ERAS in liver surgery were still based on the 2016 guideline, and multidisciplinary adherence to ERAS remained low due to the lack of a concrete, operational practice model [16].

Phase 2: Evidence-supported phase. Evidence was searched, analyzed, and synthesized to develop the guideline. A guideline development team was established, and the clinical questions and search scope were defined using the Patient, Intervention, Comparison, and Outcome framework (PICO). The literature search covered publications from 2014 to 2024 in both English and Thai. Evidence was retrieved from electronic databases including PubMed, ScienceDirect, CINAHL, and ThaiJo using predefined search terms related to hepatocellular carcinoma, hepatectomy or liver resection, ERAS protocols, practice guidelines, postoperative care, complications, and outcomes. The CPG was developed through adaptation of the ERAS Society Recommendations 2022 and synthesis of relevant evidence-based practices identified from the literature review. The ERAS Society Recommendations 2022 were selected as the primary guideline because they are the most recent and specific international recommendations for perioperative care in liver surgery. The quality of evidence was appraised using the Joanna Briggs Institute levels of evidence. Primary recommendations were derived from the Guidelines for Perioperative Care for Liver Surgery: Enhanced Recovery After Surgery (ERAS) Society Recommendations 2022, which was evaluated using the Grading of Recommendations, Assessment, Development, and Evaluation (GRADE) framework. Eighteen studies were identified, comprising Level 1a (2 studies), Level 1c (2 studies), Level 1d (1 study), Level 2c (7 studies), Level 2d (4 studies), and Level 3c (2 studies). Details of the selected recommendations are presented in Supplementary Table S1, while the search strategy, evidence appraisal, and literature search flow diagram are provided in Supplementary Table S2. Implementation feasibility was assessed, a proposed guideline was synthesized, and content validity was reviewed by experts.

Phase 3: Evidence-observed phase. The guideline was pilot tested with patients in the clinical setting. The objectives and procedures were explained to stakeholders, and personnel were trained in using the guideline and assessment tools. Outcomes were evaluated and feedback was incorporated iteratively until a practicable guideline suitable for routine use was achieved.

The developed guideline was designed to cover the care continuum across five stages: preoperative, intraoperative, postoperative, discharge planning, and post-discharge follow-up and specified physician and nursing roles and activities at each stage within a multidisciplinary care model. For implementation and evaluation, the study applied Proctor and colleagues’ framework [25], encompassing implementation outcomes (e.g., acceptability, feasibility, and appropriateness/credibility), service outcomes (e.g., efficiency and safety), and client outcomes (e.g., patient satisfaction). A multidimensional implementation strategy was employed, including educational meetings, educational materials, and audit and feedback, which were used to build a shared understanding, enhance systemic readiness, and establish a feedback loop for continuous improvement in adherence to the guidelines [25,26].

### 2.2. Sample

Purposive sampling was used based on defined inclusion/exclusion criteria. The sample consisted of: (1) eighty-four personnel using the CPG, including surgical residents and nurses from the surgical outpatient department, general/special surgical wards, surgical ICU, operating rooms, and anesthesia nurses. (2) Twenty patients with liver cancer underwent hepatectomy during the study period.

The sample size was determined by enrolling all eligible participants during the implementation period and referencing the recommendation that pilot studies should have a minimum of 12 patients [27].

The inclusion criteria included personnel who worked during the study period and were willing to participate, and patients who were diagnosed with liver cancer and underwent hepatectomy, were able to communicate in Thai or English (or had access to an interpreter), and provided informed consent. Patients aged >60 years were required to pass cognitive screening using the Mini-Cog [28].

Patients were excluded if their treatment plan was changed to another method. Personnel were excluded if they were transferred to another unit or took continuous leave during the data collection period.

#### Outcome Variables

The outcome variables were categorized according to the conceptual framework presented in Figure 1. Personnel and organizational outcomes included feasibility of CPG implementation and healthcare costs. Feasibility of CPG implementation was considered an implementation outcome, whereas healthcare costs were considered an organizational/service outcome. Patient outcomes included clinical outcomes and patient satisfaction with services. Clinical outcomes comprised postoperative complications, length of hospital stay, and 28-day readmission.

### 2.3. Implementation Instruments

#### 2.3.1. Clinical Practice Guideline for the Care of Patients with Liver Cancer Undergoing Hepatectomy

The CPG was developed through the adaptation of the ERAS Society Recommendations 2022 and synthesis of relevant evidence-based practices, covering five stages of care: (1) preoperative care (assessment; physical and psychological preparation; information provision and skill training; referral/consultation with other units as needed), (2) intraoperative care (temperature management; prevention of thromboembolism and infection; and management of tubes/lines), (3) postoperative care (pain management; early oral intake; prevention of nausea and vomiting; recovery promotion/early mobilization; early removal of tubes/lines; and surveillance for complications), (4) discharge planning (readiness assessment; wound care; warning signs; and dietary guidance), and (5) post-discharge follow-up (monitoring outcomes and ensuring follow-up treatment according to scheduled appointments). Seventeen of the 25 ERAS recommendations were selected based on clinical relevance, feasibility, resource availability, multidisciplinary applicability, and expert consensus. Details are provided in the Supplementary Table S1. The remaining eight recommendations were excluded based on multidisciplinary consensus and expert review because of limited relevance to the local healthcare context. Reasons for exclusion are provided in Supplementary Table S2. Content validity was evaluated by three experts, yielding Item-level Content Validity Index (I-CVI) and Scale-level Content Validity Index (S-CVI) of 1.00.

#### 2.3.2. Patient Handbook for Liver Cancer Patients Undergoing Hepatectomy

The handbook was developed by the researchers based on evidence and comprised two sections: (1) information about liver cancer (symptoms, risk factors, diagnosis, and treatment) and (2) self-care guidance before and after surgery, potential complications, home-care recommendations, and warning signs warranting medical attention prior to the scheduled follow-up visit. Content validity was reviewed by three experts, with an I-CVI of 1.00 and an S-CVI of 0.95.

### 2.4. Measurements

#### 2.4.1. Participant Screening Instruments

The Mini-Cognitive Assessment Instrument (Mini-Cog) in Thai was granted to use by the developer [29] and translator [28]. It comprises two parts: a three-item recall test and a clock drawing test. The total score is 0 to 5 points, with a score ≤ 3 indicating impaired cognitive function. The reported reliability coefficient is 0.86.

#### 2.4.2. Personnel Information Record Forms (Physicians and Nurses)

Developed by the researchers, including age, position, and experience in caring for patients with liver cancer undergoing hepatectomy.

#### 2.4.3. Patient Information Record Forms

Developed by the researchers, including sex, age, religion, marital status, education level, occupation, diagnosis, comorbidities, health coverage, admission date, discharge date, and treatment-related information.

#### 2.4.4. Questionnaire on the Perceived Feasibility of Implementing the Guidelines

Developed by the researchers based on the feasibility framework of Bowen and colleagues [24]. Four dimensions were assessed: acceptability, demand, implementation, and practicality across 9 items using a 5-point rating scale (total score range: 9–45). Scores were categorized into three levels using the range (class interval) method, Chusri Wongrattana (2007) [30]: 10–22 = low, 23–34 = moderate, and >35 = high. Content validity was reviewed by three experts, yielding I-CVI and S-CVI of 1.00. The questionnaire demonstrated excellent internal consistency reliability among healthcare personnel in this study, with a Cronbach’s alpha of 0.922.

#### 2.4.5. Specific Complications for Hepatectomy Assessment Form (Liver Failure, Ascites, Bile Leakage, Infection, and Bleeding System [FABIB System])

Based on the FABIB concept proposed by Li and colleagues [6], covering five complications: PHLF, ascites, bile leak, infection, and bleeding [6]. Severity was scored for each complication as none = 0, grade A = 1, grade B = 2, and grade C = 3, with a total score of 0–15. Overall severity was categorized as low (0–2), moderate (3–5), and high (≥6) [6]. Content validity was reviewed by three experts, yielding I-CVI and S-CVI of 1.00.

#### 2.4.6. CPG Compliance Record Form for Physicians and Nurses

Developed by the researchers for physicians and nurses, covering five phases of care with a total of 93 items. Adherence was assessed using structured audit based on clinical documentation and implementation records rather than self-report. Scoring: compliance = 1, non-compliance = 0 (total score range: 0–93), with higher scores indicating more complete adherence. Content validity was reviewed by three experts (I-CVI = 1.00, S-CVI = 0.98).

#### 2.4.7. Patient Satisfaction Assessment Form

The Thai version of the Short Assessment of Patient Satisfaction (SAPS) was translated by the researchers using forward and backward translation with permission from the developer [31]. The scale includes 7 items scored from 0 to 4 (total score range: 0–28), with reverse scoring for selected items. Interpretation followed the developer’s criteria: 0–10 = very dissatisfied, 11–18 = dissatisfied, 19–26 = satisfied, and 27–28 = very satisfied. Reported reliability was Cronbach’s α = 0.86, and content validity in this study was CVI = 1.00.

#### 2.4.8. Data Recording Form for the LOS, Specifying the Number of Days

Total healthcare costs were recorded as the overall inpatient costs from admission to discharge, and information on readmissions within 28 days.

### 2.5. Statistical Analysis

Data were analyzed using descriptive statistics to summarize baseline characteristics and study indicators. LOS and healthcare costs before and after CPG implementation were compared descriptively. As this feasibility-oriented study had a small patient cohort, inferential statistical testing was not performed.

## 3. Results

### 3.1. Personnel Data (Physicians and Nurses)

A total of 84 personnel participated in this study, including 8 surgical residents, 3 nurses from the surgical outpatient department, 39 nurses from the general surgical ward, 9 nurses from the special surgical ward, 14 nurses from the surgical intensive care unit (ICU), 6 operating room nurses, and 5 nurse anesthetists. The surgical residents had a mean age of 30 years (SD = 2.71), a mean duration of employment of 3 years (SD = 1.31), and a mean of 2 years of experience caring for patients undergoing hepatectomy (SD = 1.06). All residents had attended hepatectomy training (100%).

Among nurses, the ages ranged from 23 to 53 years, and work experience ranged from 1 to 35 years. The proportion of nurses who had received training related to hepatectomy ranged from 33.3% to 50%, varying by unit (OPD 33.3%, general surgical ward 38.5%, special surgical ward 33.3%, surgical ICU 42.9%, operating room 50%, and nurse anesthetists 40%), as shown in Table 1.

### 3.2. Patient Characteristics

#### 3.2.1. Patient Demographic Characteristics

A total of 20 patients were included in this study. The majority were male (75%), with ages ranging from 37 to 78 years (mean age 59 years; SD = 12.75). Most patients resided in Bangkok and the metropolitan area (90%). All were Buddhist (100%). Most were married (90%) and held a bachelor’s degree (55%). They utilized social security and universal health insurance (45%), as shown in Table 2.

#### 3.2.2. Clinical Characteristics and Surgical Data

The majority of the patients were diagnosed with hepatocellular carcinoma (55%), followed by liver metastasis (40%). A total of 85% of patients had comorbidities; the majority (90%) did not have malnutrition, and 35% had Child–Pugh grade A (the remainder did not have cirrhosis). The most common surgical approach was laparoscopic surgery (60%), and 75% underwent minor hepatectomy. Intraoperatively, 65% of patients did not receive blood products. Postoperatively, standard procedures/devices were used, including nasogastric tube placement in 65% (median duration 1 day; range 0–2 days), urinary catheterization in 100% (median 2 days; range 1–3 days), and intra-abdominal drainage in 100% (median 4 days; range 3–10 days). Regarding operative indicators, mean operative time was 181.65 ± 83.99 min, mean anesthesia time was 268.50 ± 99.78 min, and mean estimated blood loss was 765.50 ± 878.56 mL. Regarding gastrointestinal recovery, all patients resumed oral intake on postoperative day 1, with the median first flatus occurring 1 day (1–2 days) and the first defecation 2 days (1–3 days), as shown in Table 2.

### 3.3. Clinical Outcomes

All 20 participants underwent hepatectomy. Most patients (80%) experienced no postoperative complications. Four patients (20%) experienced complications, including post-hepatectomy ascites in 2 cases (10%), bile leak in 1 case (5%), and postoperative infection in 1 case (5%). Complication severity was classified as low in 3 patients (15%) and moderate in 1 patient (5%). Most patients were admitted to the intensive care unit postoperatively (85%), with a median ICU stay of 1 day. The mean length of hospital stay was 8 days (4–18 days). No readmissions occurred within 28 days. The mean total hospitalization cost was 94,280.14 THB (range 49,098–174,892 THB), as shown in Table 3.

### 3.4. Patient Satisfaction with Service Received

Patient satisfaction was assessed across seven domains. Overall satisfaction was rated as “very satisfied,” with a mean score of 27.75 ± 0.55 (26–28). All patients (100%) reported to be very satisfied with the explanation of treatment results, the care provided by healthcare personnel, their involvement in medical decision-making, the respect shown by healthcare personnel, and overall satisfaction with the care received. Regarding treatment satisfaction, 75% reported being very satisfied and 25% satisfied. Regarding the time spent with healthcare personnel, 90% reported being very satisfied and 10% satisfied, as shown in Table 4.

### 3.5. Data on Perceived Feasibility of Implementing the Clinical Practice Guideline

Perceived feasibility of implementing the CPG was assessed across four domains: acceptability, demand, implementation, and practicality (maximum score of 5 per item). Personnel reported high levels across all domains, with domain mean scores ranging from 4.27 to 4.40, indicating strong acceptance, perceived need, implementability, and practical feasibility. Negative questions were recalculated before analysis.

Considering each dimension, the mean acceptability score was 4.27 ± 0.66, and personnel indicated that the guideline should continue to be used after the study period (4.27 ± 0.63). The Demand dimension indicated that the practice guidelines were practical and relevant to the organizational context (4.29 ± 0.65), and that it was beneficial to the unit (reverse-coded item) (4.40 ± 0.68). For implementation, personnel reported that the content was easy to understand and could be applied immediately inpatient care (4.37 ± 0.58), that it provided clear care guidance (4.40 ± 0.58), and that it did not create barriers to care (reverse-coded item) (4.39 ± 0.66). For practicality, personnel reported that the guideline was easy to learn and follow (4.38 ± 0.60) and facilitated faster responses to patient reports/treatment needs (4.31 ± 0.66).

Overall, the total perceived feasibility score had a mean of 39.10 ± 4.48 (28–45). It can be concluded that the clinical guidelines are highly practical (Table 5).

Based on the feasibility assessment, personnel who used the CPG provided additional feedback through an open-ended question. Of the 84 personnel participants, eight responded to this section. Overall, the comments indicated that the CPG was appropriate and feasible for real-world use. Four respondents noted that the CPG was highly beneficial for caring for patients undergoing hepatectomy and should be continued to further improve care efficiency. However, there were also suggestions for practical improvements: two respondents recommended refining the early mobilization component because some patients were unable to fully adhere to the guideline; one respondent suggested increasing the number of electric warming blankets to meet clinical demand; and one respondent recommended shortening the assessment form to improve usability in routine practice.

### 3.6. Physician and Nursing Adherence to the Clinical Practice Guideline

Overall adherence to the CPG among physicians and nurses across all phases of care was high, indicating that the multidisciplinary team implemented almost all components. Full adherence was observed in the preoperative phase (22/22 items), while adherence in the intraoperative phase was 14 of 15 items, and in the postoperative phase was 32 of 33 items. Full adherence was also achieved in discharge planning and post-discharge follow-up (12/12 and 11/11 items, respectively). In total, 91 of 93 items were implemented, corresponding to an overall adherence rate of 97.85% (Table 6).

Items that were not fully implemented included preoperative temperature management, monitoring of INR and total bilirubin on postoperative day 5, and early postoperative mobilization. This was largely attributed to the new practice guidelines and personnel still adjusting, resource limitations, and postoperative pain, particularly among patients undergoing open surgery.

### 3.7. Comparison of Healthcare Costs and Length of Hospital Stay Before and After CPG Implementation

Comparison of healthcare costs before and after implementation of the CPG demonstrated a 50,097.88 THB (1595.47 USD) reduction in mean hospitalization costs, representing a 34.7% decrease. Additionally, minimum costs decreased by 1779.08 THB (56.66 USD; 3.5%), while maximum costs decreased by 670,518.25 THB (21,354.10 USD; 79.31%) (Table 7).

Comparison of hospital stay length before and after implementation of the CPG showed that mean LOS decreased from 14 days to 8 days, representing a 6-day reduction (42.90%). The minimum LOS decreased from 5 days to 4 days, and the maximum LOS decreased from 64 days to 18 days (Table 7).

## 4. Discussion

This study is implementation research that developed a clinical practice guideline (CPG) for patients with liver cancer undergoing hepatectomy. The guideline was adapted from the Guidelines for Perioperative Care for Liver Surgery: ERAS Society Recommendations 2022 and modified to the organizational context by selecting 17 of the 25 recommended items. The care process was structured across five phases from preoperative care through post-discharge follow-up to standardize the care pathway and reduce variability through a multidisciplinary approach. This aligns with prior evidence showing that ERAS-based liver surgery pathways reduce LOS and postoperative complications compared to traditional care [32,33,34,35].

In terms of the driving mechanism, the role of APN was as a change agent and care coordinator by synthesizing evidence, overseeing care standardization, facilitating cross-team communication, and continuously monitoring outcomes. In particular, the use of multi-component implementation strategies (educational meetings, educational materials, and audit and feedback) is supported by evidence demonstrating significant improvements in guideline adherence [26]. These findings are consistent with reports that higher adherence is a key predictor of improved patient outcomes [33,36], including reduced variability in care when clear procedures are established [37].

The majority of patients in the study were male with an average age of 59 years, which is consistent with the epidemiology of liver cancer, which is more common in men and middle-aged-to-older adults. Most patients had preserved liver function and no malnutrition, consistent with the criteria for surgical suitability and the ERAS principle emphasizing preoperative optimization [38,39]. In addition, the relatively high proportion of laparoscopic hepatectomy reflects the growing trend toward minimally invasive surgery, which has been associated with faster postoperative recovery in the literature [40].

The clinical outcomes showed favorable trends following CPG implementation; however, they should be interpreted cautiously. The post-implementation period showed a lower observed complication rate, shorter length of stay, and lower healthcare costs than the historical comparison period, without 28-day readmissions. Nevertheless, differences in length of stay and healthcare costs were based on historical pre-implementation data and were not adjusted for baseline characteristics or case mix. Therefore, these findings cannot be attributed directly to the CPG implementation. Alternative explanations, including differences in patient characteristics, surgical approach, institutional experience, resource use, and other contextual factors, may have contributed to the observed differences. These findings are consistent with previous ERAS evidence [11,41]. Nevertheless, previous studies also indicate that ERAS implementation is complex and may be affected by local resources, team engagement, adherence monitoring, and workflow barriers. Given the small patient cohort, historical comparison, potential confounding, and absence of inferential testing, the findings should be viewed as preliminary descriptive signals rather than evidence of effectiveness. Patient satisfaction was also high, consistent with patient-centered care principles [42,43,44,45]. The short implementation period limited the evaluation of long-term sustainability, underscoring the need for a longer follow-up period.

In terms of acceptability and feasibility, personnel perceived a high level of feasibility for implementing the guideline across the domains of acceptability, demand, implementation, and practicality. Together with the high adherence rate, these implementation outcomes represent the main findings of this feasibility-oriented study and align with the study objectives. This finding is consistent with Bowen et al.’s feasibility framework and Proctor et al.’s implementation outcomes [24,25]. The findings also suggest that evidence-based guidelines developed with consideration of the local context and implemented through structured, multi-component strategies may be acceptable and practical for routine clinical use, consistent with previous guideline implementation research [46].

However, three main practical gaps representing opportunities for improvement were identified: first, preoperative body temperature management was a newly introduced component, resulting in incomplete adherence during the initial implementation phase. This finding is consistent with the literature on barriers to practice change, which suggests that compliance typically improves with structured education and ongoing monitoring [47]. Second, surveillance for PHLF based on the ISGLS criteria on postoperative day (POD) 5 remained variable and dependent on clinical judgment, despite ISGLS recommendations emphasizing INR and bilirubin levels on POD5 as core diagnostic parameters [1]. New evidence suggests that monitoring from POD3 may allow for earlier risk screening [48]. Third, early mobilization was not fully achieved in some patients, particularly those undergoing open surgery who experienced significant postoperative pain. This highlights the need for proactive, multimodal pain management strategies to promote early mobilization in accordance with ERAS principles [49]. This finding aligns with evidence demonstrating that early mobilization reduces postoperative complications and length of hospital stay [50,51], as well as with contemporary concepts of pain management integrating both pharmacological and non-pharmacological interventions [52,53].

In summary, this study indicates that developing a clinical practice guideline grounded in the ERAS 2022 [11]. recommendations and operationalized through multi-component implementation strategies, with APN competencies as a key implementation driver, was feasible, acceptable, and associated with high adherence in routine practice. Clinical outcomes showed favorable exploratory trends.

### 4.1. Implication for Practice

The findings from this study indicate that the CPG was feasible to use. To ensure sustainable implementation, hospitals should establish a structured support system with clearly assigned unit leads to oversee, monitor, and evaluate adherence, alongside ongoing on-the-job training to strengthen personnel capability and context-specific adaptation. Practical tools, such as concise, trackable care pathway diagrams and brief guideline summaries specifying key roles and actions at each phase, should be developed to support consistent multidisciplinary practice. In the future, a digital patient tracking system should be implemented to enable continuous care from admission through post-discharge follow-up by facilitating real-time information sharing, symptom monitoring, complication reporting, and recovery assessment. Future research should include larger cohorts, longer-term outcomes (e.g., post-discharge quality of life), and analysis of barriers and facilitators to adherence to inform scale-up to other hospital settings.

### 4.2. Strengths and Limitations

The strength of this study lies in the systematic development and real-world implementation of the CPG based on Soukup’s evidence-based practice model and Proctor and colleagues’ implementation outcomes framework. However, the study included a small patient cohort and used historical data only for descriptive comparison of length of stay and hospitalization costs. In addition, although the primary recommendations were derived from the ERAS Society Recommendations 2022, which were evaluated using the GRADE framework, the final CPG developed for the local context was reviewed by experts for content validity rather than formally evaluated using GRADE by an expert panel. Because detailed baseline and case-mix data were limited, the findings should not be interpreted as causal evidence. Potential differences in patient characteristics, surgical approach, team experience, staff composition, institutional expertise, selection bias, and observation-related bias may have influenced the findings. Awareness of protocol implementation and monitoring may have introduced performance bias, potentially overestimating compliance and outcomes. In addition, the high satisfaction and feasibility scores may reflect ceiling effects, which could limit the ability to detect variation in patient and personnel perceptions. The short implementation period also limited assessment of sustainability; therefore, longer follow-up, ongoing training, and periodic audit and feedback are needed.

## 5. Conclusions

This study found that the developed CPG was feasible and contextually appropriate for ERAS-based hepatectomy care in this setting. Healthcare personnel demonstrated high adherence to the guideline and reported high perceived feasibility across the domains of acceptability, demand, implementation, and practicality. These implementation outcomes represent the main findings of this feasibility-oriented study. Favorable exploratory trends were observed in selected patient and service outcomes, including postoperative complications, recovery indicators, length of hospital stay, healthcare costs, and patient satisfaction. However, these clinical outcomes should be interpreted cautiously because of the small patient cohort, historical comparison, lack of inferential testing, and potential confounding factors. Larger controlled studies with longer follow-up are needed to determine effectiveness and sustainability.

## Figures and Tables

**Table 1:** Demographic characteristics of the participants (N = 84).

Personnel Group	N	Age (Years), Mean ± SD (Range)	Work Experience, N (Percent)	Hepatectomy Training, n (Percent)
surgical residents	8	30.25 ± 2.71 (27–35)	3–4 years, 4 (50.00)	8 (100.0)
surgical outpatient ward nurses	3	45.00 ± 6.80 (45–50)	>10 years, 3 (100.00)	1 (33.30)
general surgical inpatient ward nurses	39	29.62 ± 7.30 (23–53)	1–5 years, 22 (56.40)	15 (38.50)
specialized surgical inpatient ward nurses	9	31.22 ± 8.18 (23–45)	1–5 years, 5 (55.60)	3 (33.30)
surgical critical care nurses	14	30.70 ± 7.06 (24–46)	1–5 years, 10 (78.60)	8 (57.10)
operating room nurses	6	35.00 ± 6.41 (29–45)	6–10 years, 3 (50.00); >10 years, 3 (50.00)	2 (33.30)
anesthesia nurses	5	33.60 ± 4.39 (28–40)	1–5 years, 4 (80.00)	2 (40.00)

Values are presented as N (Percent) or mean ± SD (range). For work experience, the most common category in each personnel group is reported to condense the table.

**Table 2:** Patient demographic, clinical, surgical, and postoperative recovery characteristics (N = 20).

Characteristics	n (Percent)
**Demographic characteristics**	
Male sex	15 (75.00)
Age (years), mean ± SD (range) 59.00 ± 12.75 (37–78)	
Bangkok/metropolitan residence	18 (90.00)
Married	18 (90.00)
Bachelor’s degree or higher	12 (60.00)
Social security/universal coverage scheme	18 (90.00)
**Clinical characteristics**	
Diagnosis: HCC/liver metastasis/intrahepatic cholangiocarcinoma	11 (55.00)/8 (40.00)/1 (5.00)
Comorbidities	17 (85.00)
Nutritional status: NT-1	18 (90.00)
Child–Pugh class A/Not applicable (no cirrhosis)	7 (35.00)/13 (65.0)
**Surgical characteristics**	
Surgical approach: laparoscopic/open	12 (60.0)/8 (40.0)
Extent of hepatectomy: minor/major	15 (75.0)/5 (25.0)
Intraoperative blood transfusion	7 (35.0)
Operative time (min), mean ± SD (range) 181.65 ± 83.99 (85–340)	
Anesthesia time (min), mean ± SD (range) 268.50 ± 99.78 (150–450)	
Estimated blood loss (mL), mean ± SD (range) 765.50 ± 878.56 (10–2800)	
**Postoperative recovery characteristics**	
Nasogastric tube placement/duration (days), median (range) 13 (65.0)/1 (0–2)	
Urinary catheterization/duration (days), median (range) 20 (100.0)/2 (1–3)	
Abdominal drainage/duration (days), median (range) 20 (100.0)/4 (3–10)	
Time to oral intake (days), mean ± SD1.00 ± 0.00	
Time to first flatus (days), median (range) 1 (1–2)	
Time to first defecation (days), median (range) 2 (1–3)	

Values are presented as N (Percent), mean ± SD (range), or median (range), as appropriate.

**Table 3:** Clinical outcomes (N = 20).

Clinical Outcomes	n (Percent)
**Postoperative complications**	
Any complication	4 (20.0)
No complication	16 (80.0)
Post-hepatectomy liver failure	0 (0.0)
Post-hepatectomy ascites	2 (10.0)
Bile leakage	1 (5.0)
Postoperative infection	1 (5.0)
Bleeding	0 (0.0)
**Complication severity**	
Low	3 (15.0)
Moderate	1 (5.0)
Readmission within 28 days	0 (0.00)
ICU admission	17 (85.0)
ICU/Semi-ICU length of stay (days), median (range) 1 (0–1)	
Hospital length of stay (days), mean ± SD (range) 8.00 ± 7.00 (4–18)	
Hospitalization cost (THB), mean ± SD (range) 94,280.14 ± 49,098.00 (49,098–174,892)	

**Table 4:** Patient satisfaction with the service received.

Patient Satisfaction with the Service Received	Min–Max	M	SD	Very Satisfied
				n (Percent)
1. Treatment satisfaction	3–4	3.85	0.36	15 (75)
2. Explanation of treatment results	4–4	4.00	0.00	20 (100)
3. Clinician care	4–4	4.00	0.00	20 (100)
4. Participation in medical decision-making	4–4	4.00	0.00	20 (100)
5. Respect by the clinician	4–4	4.00	0.00	20 (100)
6. Time with the clinician	3–4	3.90	0.31	18 (90)
7. Satisfaction with hospital/clinic care	4–4	4.00	0.00	20 (100)
**Overall satisfaction**	26–28	27.75	0.55	

**Table 5:** Perceived feasibility of implementing the CPG.

Content	Recognizing the Feasibility of Implementing the Practice			
	Min–Max	M	SD	Feasibility Level, n (Percent)
1. The content of CPG is easy to understand, enabling care to be provided immediately according to this practice. (Implementation)	3–5	4.37	0.58	High, 80 (95.2)
2. CPG, suitable for the situation, and aligned with the organization’s culture. (Demand)	3–5	4.29	0.65	High, 75 (89.3)
3. CPG may offer less benefits to agencies or organizations. (Demand)	3–5	4.40	0.68	High, 75 (89.3)
4. CPG recommends continuous application, even after the study concludes. (Acceptability)	3–5	4.27	0.66	High, 74 (88.1)
5. Personnel find satisfaction in CPG. (Acceptability)	3–5	4.27	0.63	High, 76 (90.5)
6. This CPG establishes a standardized approach to caring for patients with liver cancer undergoing hepatectomy. (Implementation)	3–5	4.40	0.58	High, 80 (95.2)
7. Using this guideline creates barriers to my ability to care for patients with liver cancer undergoing hepatectomy. (Implementation)	3–5	4.39	0.66	High, 76 (90.5)
8. You can learn and adhere to CPG. (Practicality)	3–5	4.38	0.60	High, 79 (94.0)
9. This CPG enable patients to receive quicker responses to reporting and treatment. (Practicality)	3–5	4.31	0.66	High, 75 (89.3)
**Total**	28–45	39.10	4.48	High, 71 (84.5)

**Table 6:** Physician and nursing adherence to the clinical practice guideline.

Phase of Care	Items Implemented /Total Items (Percent)
Preoperative phase	22/22 (100.00)
Intraoperative phase	14/15 (93.30)
Postoperative phase	32/33 (97.00)
Discharge-planning phase	12/12 (100.00)
Post-discharge follow-up phase	11/11 (100.00)
**Overall**	**91/93 (97.80)**

**Table 7:** Descriptive comparison of healthcare costs and length of hospital stay before and after CPG implementation.

Period	Healthcare Costs		LOS Mean (Min–Max) (Days)
	Mean/Median (Min–Max) (THB)	Mean/Median (Min–Max) (USD)	
Pre-implementation period (2020–2024; N = 360)	144,378.02/98,747.50 (50,877.08–845,410.25)	4598.03/3000.99 (1620.29–26,923.91)	14 (5–64)
CPG implementation period (March–July 2025; N = 20)	94,280.14/81,926.38 (49,098.00–174,892.00)	3002.55/2489.79 (1563.63–5569.81)	8 (4–18)

## Data Availability

The data presented in this study are available on request from the corresponding author to protect the privacy of the research participants.

## Supplementary Materials

The following supporting information can be downloaded at https://www.mdpi.com/article/10.3390/healthcare14131939/s1, Table S1: Recommendations of the clinical practice guideline (CPG) for patients undergoing hepatectomy. Table S2: ERAS 2022 recommendations not included in the CPG. Refs. [6,10,11,54,55,56,57,58,59,60,61,62,63,64,65,66,67,68,69,70,71,72,73,74,75,76,77,78,79,80,81,82,83,84,85] are cited in the Supplementary Material file.

## Abbreviations

The following abbreviations are used in this manuscript:

CPG	Clinical practice guideline
ERAS	Enhanced Recovery After Surgery
APN	Advanced practice nurse
