# Supplementary material for: Development and Evaluation of Clinical Practice Guidelines for Patients Undergoing Hepatectomy
Source: Healthcare (Basel). 2026 Jul 1;14(13):1939. doi: 10.3390/healthcare14131939 (PMC13362505; doi:10.3390/healthcare14131939)
Supplement: Supplementary file 1 [file healthcare-14-01939-s001.zip › healthcare-4322351-supplementary.pdf]

Supplementary Table S1

Recommendations of the clinical practice guideline (CPG) for patients undergoing hepatectomy

This CPG covers 5 stages of patient care, including preoperative, intraoperative, postoperative, discharge planning, and post-discharge.

Stage	Recommendations	Physician's activities	Nurse's activities
Preoperative	1. Preoperative counseling (ERAS 2022, Weak Recommend)	Provide patients with essential information regarding the disease, treatment options, indications for hepatectomy, and potential complications (1) (level 1a), obtain informed consent, and arrange appropriate consultations with an internist, anesthesiologist, and other relevant specialists as indicated (2) (level 1a).Patients should also be informed of procedural costs based on healthcare coverage, including any potential out-of-pocket expenses.	Provide concise education on the disease and its treatment, distribute a patient handbook for individuals undergoing hepatectomy, and offer counseling to patients and families with opportunities for questions (1) (level 1a). Informed consent documentation should be verified, healthcare coverage and referral requirements clarified, and treatment costs and financial capacity assessed, with referral to social services when indicated.
	2. Prehabilitation counseling (ERAS 2022, Weak Recommend)	Assess indications for hepatectomy, review radiological imaging (MRI), and evaluate patient risk factors, comorbidities, and liver function (3) (level 1a). Evaluate physical status with essential laboratory tests (CBC, BUN, Cr, Electrolyte, Coagulogram, LFT, BS, anti-HIV) and imaging (Chest X-ray, EKG), issue standardized	Assess the patient's signs and symptoms, risk factors, and comorbidities (3) (level 1a), perform laboratory investigations as prescribed, and evaluate psychosocial status while addressing patient concerns (4) (level 3c). Family involvement in care should also be encouraged. Provide pre- and postoperative

Stage	Recommendations	Physician's activities	Nurse's activities
		preoperative orders for hepatectomy, provide pre- and postoperative education, review current medications and those to be withheld before surgery, and ensure readiness of the intensive or high-dependency care unit.	education on hepatectomy, including breathing exercises, effective coughing, use of incentive spirometry (Triflow), postoperative pain management, shared goal setting for early mobilization, and preoperative antiseptic bathing on the night before and the morning of surgery, along with offering emotional support to encourage patient confidence and coping.
	3. Preoperative biliary drainage (ERAS 2022, Strong Recommend)	Assess the need for preoperative biliary drainage in patients with cholestatic liver disease (>3mg/dl)	N/A
	4. Preoperative smoking and alcohol cessation (ERAS 2022, Strong Recommend)	Advise implementing preoperative lifestyle modification, including smoking cessation for at least 4 weeks and alcohol abstinence for 4–8 weeks before surgery (5, 6) (level 1b).	Advise smoking and alcohol cessation and to guide healthy behavior modification.
	5. Preoperative nutrition (ERAS 2022, Strong Recommend)	Assess patients' nutritional status prior to surgery (7, 8) (level 2d, 2a), and to correct malnutrition before hepatectomy when identified.	Assess nutritional status using the Nutrition Note tool (7, 8) (level 2d, 2a).

Stage	Recommendations	Physician's activities	Nurse's activities
	6. Minimally invasive surgery (ERAS 2022, Strong Recommend)	Consider laparoscopic hepatectomy when feasible (9) (level 1a).	N/A
Intraoperative	7. Anti-thrombotic prophylaxis (ERAS 2022, Strong Recommend)	Use intermittent pneumatic compression devices.	Monitor the function of the pneumatic compression device. (10, 11) (level 1c)
	8. Antimicrobial prophylaxis and skin preparation (ERAS 2022, Weak Recommend)	Advise an antiseptic shower on the night before surgery and again on the morning of surgery. (12) (level 1c) The order is to administer antibiotics 30 to 60 minutes before surgery. (13, 14) (level 1a)	Advise an antiseptic shower on the night before surgery and again on the morning of surgery. (12) (level 1c) Prophylactic antibiotics are administered 30–60 minutes before surgery. (13, 14) (level 1a)
	9. Preventing intraoperative hypothermia (ERAS 2022, Strong Recommend)	Manage the patient's body temperature according to the Clinical Guideline for the Prevention and Management of Perioperative Hypothermia. (15)(level 2d)	Manage the patient's body temperature in accordance with the Clinical Guideline for the Prevention and Management of Perioperative Hypothermia (15) (Level 2d). Activities are organized into three phases: the preoperative phase (ward nurse), intraoperative phase (operating room nurse and nurse anesthetist), and postoperative phase (post-anesthesia care

Stage	Recommendations	Physician's activities	Nurse's activities
			unit nurse and the receiving ward nurse after surgery).
postoperative	10. Prophylactic nasogastric intubation (ERAS 2022, Strong Recommend)	Consider use only when necessary and remove before emergence from anesthesia or as soon as possible after surgery (16, 17) (Level 1b, 2d).	N/A
	11. Prophylactic abdominal drainage (ERAS 2022, Strong Recommend)	Consider use only when necessary. (9) (level 1a)	N/A
	12. Epidural, postoperative intravenous, and postoperative per oral analgesia (ERAS 2022, Strong Recommend)	Pain management is delivered in accordance with the hospital's postoperative pain management guidelines. Assessed for pain control by the anesthesia team, with pain evaluated using the Numeric Rating Scale (NRS), and adjunct analgesics prescribed as appropriate.	Assess pain using the Numeric Rating Scale (NRS), observe facial expressions and behavior, and document pain findings continuously. Provide pain management using both pharmacological and non-pharmacological approaches (18, 19) (Level 2c, 2a). For pharmacological management, administer analgesics, reassess pain after administration to ensure effective control, and monitor for analgesic-related adverse effects. For non-pharmacological management, identify the patient's preferred activities and recommend

Stage	Recommendations	Physician's activities	Nurse's activities
			distraction strategies (e.g., reading, meditation), teach and practice relaxation and breathing exercises, provide information and emotional support, maintain a comfortable position, and optimize the environment (cleanliness, lighting, and appropriate temperature) to promote relaxation and reduce pain. Encourage family members to provide support to reduce stress and pain triggers and assess for postoperative delirium.
	13. Postoperative artificial nutrition and early oral intake (ERAS 2022, Strong Recommend)	Order sips of water 6 hours after surgery, start a liquid diet on postoperative day 1, and advance to a regular diet as tolerated on postoperative day 2 (7, 20) (Level 2d, 1c).	Ensure the patient follows the prescribed diet plan and assess for symptoms and clinical signs after meals.
	14. Postoperative glycemic control (ERAS 2022, Strong Recommend)	Monitor postoperative blood glucose every 6 hours for 24 hours, maintaining levels between 80–180 mg/dL. Provide glucose-lowering therapy as indicated; for patients with diabetes or uncontrolled hyperglycemia, consider	Monitor postoperative blood glucose every 6 hours for 24 hours, maintaining levels between 80–180 mg/dL.

Stage	Recommendations	Physician's activities	Nurse's activities
		consultation with an endocrinologist for co-management (21) (Level 2c).	
	15. Stimulation of bowel movement (ERAS 2022, Weak Recommend)	Assess bowel function by monitoring bowel sounds, flatus, and bowel movements, and consider prescribing laxatives as indicated (22, 23) (Level 1c).	Assess bowel function by monitoring bowel sounds, flatus, and bowel movements. (22, 23) (Level 1c).
	16.Early and scheduled mobilization (ERAS 2022, Strong Recommend)	Advise the patient to begin postoperative mobilization as early as possible.	Promote early postoperative mobilization (24–28) (Level 1b, 2c): on postoperative day 1, assist the patient to sit on the bed 3–4 times/day for 15 minutes each; on postoperative day 1, ensure sitting on the bed 3–4 times/day for 15 minutes each and standing at the bedside; on postoperative day 2, encourage walking >100–200 meters 2–3 times/day; and on postoperative day 3, encourage walking 250–300 meters more than 5 times/day. Encourage use of an incentive spirometer (Triflow) from postoperative day 1 and perform breathing exercises for 5 minutes per session at least 4 times/day (29, 30) (Level 1b, 1c).

Stage	Recommendations	Physician's activities	Nurse's activities
	17.Postoperative nausea and vomiting (PONV) prophylaxis (ERAS 2022, Strong Recommend)	Administer prophylactic antiemetics to prevent postoperative nausea and vomiting (31) (Level 2a).	Assess postoperative nausea and vomiting and administer antiemetics according to the treatment plan.
	Monitoring of specific postoperative complications (FABIB System) (Evidence-based) (32) (level 2b)	On postoperative day (POD) 1, monitor post-hepatectomy hemorrhage (PHH) by ordering a CBC and grading severity using the FABIB system. On POD 3, assess for infection (consider antibiotics per institutional standards, monitor temperature >38°C or <36°C, inspect the wound and drain sites, review urinary catheter necessity, and follow CBC/cultures). Assess for bile leak with drain-fluid bilirubin (if bile-stained) and serum bilirubin. Assess post-hepatectomy ascites using the FABIB system. On POD 5, assess post-hepatectomy liver failure (PHLF) with INR and total serum bilirubin.	Monitor PHH with CBC on ward arrival and again on POD 1, hourly vital signs and oxygen saturation with prompt reporting of abnormalities, trend labs. On POD 3, assess infection by inspecting the wound and drain sites, monitoring temperature (>38°C or <36°C), administering antibiotics per institutional standards, assessing urine characteristics if a catheter is in place. Assess bile leak with drain-fluid and serum bilirubin (report if drain-fluid bilirubin >3× serum). Assess post-hepatectomy ascites by documenting drain output (report >500 mL/day) or assessing abdominal distension if no drain. On POD 5, assess post-hepatectomy liver failure (PHLF) by obtaining and trending labs,

Stage	Recommendations	Physician's activities	Nurse's activities
			reporting abnormalities, and grading severity using the FABIB system.
Discharge planning	Prepare the patient for discharge (Evidence-based) (33) (level 3c)	Assess the patient's condition and readiness for discharge, provide instructions on wound care and warning signs that require medical attention, arrange follow-up appointments, and assess anxiety in the patient and family.	Assess the patient's clinical status (wound and complications), self-care ability (ADLs, mobility, oral intake, and elimination), and family/caregiver readiness, and promote family involvement. Provide discharge education on wound care, correct medication use, a postoperative diet (easy-to-digest, high-protein, low-fat), and gradual physical rehabilitation (e.g., walking and stretching). Assess psychological status, including anxiety or depression (34) (Level 2c), and address individual concerns with tailored counseling.
Post-discharge	Continuity of care follow-up. (Evidence-based) (33) (level 3c)	Arrange a follow-up visit at the surgical outpatient clinic one week after discharge, assess the surgical wound, monitor liver function using laboratory results, and provide guidance on long-term follow-up surveillance.	At the surgical outpatient clinic, assess symptoms, the surgical wound, and liver function based on laboratory results, and review self-care and home-related problems with individualized advice. Reinforce long-term follow-up every 3–6 months for the first 2 years

Stage	Recommendations	Physician's activities	Nurse's activities
			after surgery and every 6–12 months thereafter to monitor liver function, recovery, and cancer recurrence surveillance (35) (Level 2b).

Note. Level of evidence

Level 1 – Experimental Designs

Level 1.a Systematic Review of Randomized Controlled Trials (RCT)

Level 1.b Systematic Review of RCTs and Other Study Designs

Level 1.c RCT

Level 1.d Pseudo-RCTs

Level 2 – Quasi- Experimental Designs

Level 2.a Systematic Review of Quasi- Experimental Studies

Level 2.b Systematic Review of Quasi- Experimental and Other Lower Study Designs

Level 2.c Quasi-Experimental Prospectively Controlled Study

Level 2.d Pre-test-Post-test or Historic/Retrospective Control Group Study

Level 3 – Observational – Analytic Design

Level 3.a Systematic Review of Comparable Cohort Studies

Level 3.b Systematic Review of Comparable Cohort and Other Lower Study Designs

Level 3.c Cohort Study with Control Group

Level 3.d Case-Controlled Study

Level 3.e Observational Study Without a Control Group

Reference

1. Brodersen F, Wagner J, Uzunoglu FG, Petersen-Ewert C. Impact of Preoperative Patient Education on Postoperative Recovery in Abdominal Surgery: A Systematic Review. World J Surg. 2023;47(4):937-47.
2. Pham CT, Gibb CL, Fitridge RA, Karnon JD. Effectiveness of preoperative medical consultations by internal medicine physicians: a systematic review. BMJ Open. 2017;7(12):e018632.
3. Dewulf M, Verrips M, Coolsen MME, Olde Damink SWM, Den Dulk M, Bongers BC, et al. The effect of prehabilitation on postoperative complications and postoperative hospital stay in hepatopancreatobiliary surgery a systematic review. HPB (Oxford). 2021;23(9):1299-310.
4. Kassahun WT, Mehdorn M, Wagner TC, Babel J, Danker H, Gockel I. The effect of preoperative patient-reported anxiety on morbidity and mortality outcomes in patients undergoing major general surgery. Scientific Reports. 2022;12(1):6312.
5. Alsanad, M.; Aljanoubi, M.; Alenezi, F.K.; Farley, A.; Naidu, B.; Yeung, J. Preoperative smoking cessation interventions: A systematic review and meta-analysis. Perioper. Med. 2025, 14, 5. https://doi.org/10.1186/s13741-024-00479-4.
6. Egholm JW, Pedersen B, Moller AM, Adami J, Juhl CB, Tonnesen H. Perioperative alcohol cessation intervention for postoperative complications. Cochrane Database Syst Rev. 2018;11(11):CD008343.
7. Fujio A, Miyagi S, Tokodai K, Nakanishi W, Nishimura R, Mitsui K, et al. Effects of a new perioperative enhanced recovery after surgery protocol in hepatectomy for hepatocellular carcinoma. Surg Today. 2020;50(6):615-22.
8. Fan X, Chen G, Li Y, Shi Z, He L, Zhou D, Lin H. The Preoperative Prognostic Nutritional Index in Hepatocellular Carcinoma After Curative Hepatectomy: A Retrospective Cohort Study and Meta-Analysis. J Invest Surg. 2021;34(8):826-33.
9. Joliat GR, Kobayashi K, Hasegawa K, Thomson JE, Padbury R, Scott M, et al. Guidelines for Perioperative Care for Liver Surgery: Enhanced Recovery After Surgery (ERAS) Society Recommendations 2022. World J Surg. 2023;47(1):11-34.
10. Ho KM, Tan JA. Stratified meta-analysis of intermittent pneumatic compression of the lower limbs to prevent venous thromboembolism in hospitalized patients. Circulation. 2013;128(9):1003-20.

11. Lobastov K, Sautina E, Alencheva E, Bargandzhiya A, Schastlivtsev I, Barinov V, et al. Intermittent Pneumatic Compression in Addition to Standard Prophylaxis of Postoperative Venous Thromboembolism in Extremely High-risk Patients (IPC SUPER): A Randomized Controlled Trial. Ann Surg. 2021;274(1):63-9.
12. Hsieh CS, Cheng HC, Lin JS, Kuo SJ, Chen YL. Effect of 4% chlorhexidine gluconate predisinfection skin scrub prior to hepatectomy: a double-blinded, randomized control study. Int Surg. 2014;99(6):787-94.
13. Steccanella F, Amoretti P, Barbieri MR, Bellomo F, Puzziello A. Antibiotic Prophylaxis for Hepato-Biliopancreatic Surgery-A Systematic Review. Antibiotics (Basel). 2022;11(2).
14. Sartelli M, Coccolini F, Labricciosa FM, Al Omari AH, Bains L, Baraket O, et al. Surgical Antibiotic Prophylaxis: A Proposal for a Global Evidence-Based Bundle. Antibiotics (Basel). 2024;13(1).
15. Clinical guideline for prevention and management of perioperative hypothermia [Internet]. 2023. Available from: https://www.rcat.org/_files/ugd/09122c_973768a8cc2a438d91b5ecbf8428b6fc.pdf?lang=en.
16. Wen Z, Zhang X, Liu Y, Bian L, Chen J, Wei L. Is routine nasogastric decompression after hepatic surgery necessary? A systematic review and meta-analysis. (1873-491X (Electronic)).
17. Arfa S, Turco C, Lakkis Z, Bourgeois S, Fouet I, Evrard P, et al. Delayed return of gastrointestinal function after hepatectomy in an ERAS program: incidence and risk factors. HPB (Oxford). 2022;24(9):1560-8.
18. Dieu AA-O, Huynen P, Lavand'homme P, Beloeil H, Freys SM, Pogatzki-Zahn EM, et al. Pain management after open liver resection: Procedure-Specific Postoperative Pain Management (PROSPECT) recommendations. (1532-8651 (Electronic)).
19. Joshi GP, Kehlet H. Postoperative pain management in the era of ERAS: An overview. (1878-1608 (Electronic)).
20. Feng J, Xu R, Li K, Li F, Gao M, Han Q, et al. Effects of preoperative oral carbohydrate administration combined with postoperative early oral intake in elderly patients undergoing hepatectomy with acute-phase inflammation and subjective symptom burden: A prospective randomized controlled study. (0219-3108 (Electronic)).

21. Blixt C, Larsson M, Isaksson B, Ljungqvist O, Rooyackers O. The effect of glucose control in liver surgery on glucose kinetics and insulin resistance. Clin Nutr. 2021;40(7):4526-34.

22. van Woerden V, Olij B, Fichtinger RS, Lodewick TM, Coolsen MME, Den Dulk M, et al. The orange-III study: the use of preoperative laxatives prior to liver surgery in an enhanced recovery programme, a randomized controlled trial. HPB. 2022;24(9):1492-500.

23. Hendry PO, van Dam RM, Bukkems SF, McKeown DW, Parks RW, Preston T, et al. Randomized clinical trial of laxatives and oral nutritional supplements within an enhanced recovery after surgery protocol following liver resection. Br J Surg. 2010;97(8):1198-206.

24. de Almeida EPM, de Almeida JP, Landoni G, Galas F, Fukushima JT, Fominskiy E, et al. Early mobilization programme improves functional capacity after major abdominal cancer surgery: a randomized controlled trial. Br J Anaesth. 2017;119(5):900-7.

25. Ni CY, Wang ZH, Huang ZP, Zhou H, Fu LJ, Cai H, et al. Early enforced mobilization after liver resection: A prospective randomized controlled trial. Int J Surg. 2018;54(Pt A):254-8.

26. Burgess LC, Immins T, Wainwright TW. What is the role of post-operative physiotherapy in general surgical Enhanced Recovery after Surgery pathways? European Journal of Physiotherapy. 2019;21(2):67-72.

27. Tazreean R, Nelson G, Twomey R. Early mobilization in enhanced recovery after surgery pathways: current evidence and recent advancements. Journal of Comparative Effectiveness Research. 2022;11(2):121-9.

28. Li Z, Zhou L, Li M, Wang W, Wang L, Dong W, et al. Early mobilization after pancreatic surgery: A randomized controlled trial. Surgery. 2024;176(4):1179-88.

29. Chang P-C, Chen P-H, Chang T-H, Chen K-H, Jhou H-J, Chou S-H, Chang T-W. Incentive spirometry is an effective strategy to improve the quality of postoperative care in patients. Asian Journal of Surgery. 2023;46(9):3397-404.

30. Zhao CH, Sun YH, Mao XM. Volume Incentive Spirometry Reduces Pulmonary Complications in Patients After Open Abdominal Surgery: A Randomized Clinical Trial. 2023(1178-7074 (Print)).

31. Schwartz J, Gan TJ. Management of postoperative nausea and vomiting in the context of an Enhanced Recovery after Surgery program. Best Practice & Research Clinical Anaesthesiology. 2020;34(4):687-700.

32. Li J, Moustafa M, Freiwald-Bibiza E, Alzudjali A, Fischer L, Nashan B. Is It Feasible to Standardize a Composite Postoperative Complication Reporting System for Liver Resection? Journal of Gastrointestinal Surgery. 2020;24(12):2748-55.

33. Yuan Y. Impact of Multidisciplinary Continuity of Care on Postoperative Outcomes in Liver Cancer Surgical Patients. J Multidiscip Healthc. 2025; 18:4749–59.

34. Lee H-H, Chiu C-C, Lin J-J, Wang J-J, Lee K-T, Sun D-P, Shi H-Y. Impact of preoperative anxiety and depression on quality of life before and after resection of hepatocellular carcinoma. Journal of Affective Disorders. 2019; 246:361-7.

35. National Comprehensive Cancer Network (NCCN). Hepatocellular Carcinoma (Version 3.2024) [Internet]. NCCN Clinical Practice Guidelines in Oncology. 2024 [cited 2024 Oct 7]. Available from: https://www.nccn.org/guidelines/guidelines-detail?category=1&id=1411

Supplementary Table S2

ERAS 2022 recommendations not included in the CPG

Items	Reason for exclusion
1. Perioperative oral immunonutrition	Not included because ERAS 2022 does not currently recommend routine immunonutrition in hepatic surgery due to limited evidence. Nutritional assessment and correction of malnutrition were already included under preoperative nutrition.
2. Preoperative fasting and preoperative carbohydrate loading	Not included as a separate CPG item because preoperative fasting was already managed according to existing anesthetic practice, and carbohydrate loading was not routinely implemented in the local context during the pilot period.
3.Pre-anesthetic medication	Not included as a separate CPG item because pre-anesthetic medication decisions were already individualized and managed by the anesthesia team according to existing institutional practice.
4. Preoperative steroid administration	Not included because routine steroid administration was not part of existing local perioperative practice and required further multidisciplinary agreement, particularly for patients with diabetes or other contraindications.
5. Wound catheter and transversus abdominis plane block	Not included because regional analgesic techniques were not routinely feasible for all patients and depended on anesthesiologist assessment, surgical approach, coagulation status, and patient-specific contraindications.
6. Prevention of delayed gastric emptying	Not included because this recommendation is mainly relevant to selected procedures such as left-sided liver resection and was not broadly applicable to all patients undergoing hepatectomy in this protocol.

Items	Reason for exclusion
7. Fluid management	Not included as a separate CPG item because intraoperative fluid management was already managed by the anesthetic team according to patient condition and surgical requirements.
8. Monitoring/audit	Not included as a direct patient-care recommendation in the CPG protocol because audit and feedback were incorporated as part of the implementation strategy and outcome evaluation process.
